# Identification and functional interpretation of miRNAs affected by rare CNVs in CAKUT

**DOI:** 10.1038/s41598-022-22749-1

**Published:** 2022-10-22

**Authors:** Kristina Mitrovic, Ivan Zivotic, Ivana Kolic, Ana Djordjevic, Jelena Zakula, Jelena Filipovic Trickovic, Maja Zivkovic, Aleksandra Stankovic, Ivan Jovanovic

**Affiliations:** 1 Department of Radiobiology and Molecular Genetics, “Vinča” Institute of Nuclear Sciences, National Institute of the Republic of Serbia, University of Belgrade, Belgrade, Serbia; 2 Department of Molecular Biology and Endocrinology, “Vinča” Institute of Nuclear Sciences, National Institute of the Republic of Serbia, University of Belgrade, Belgrade, Serbia; 3 Department of Physical Chemistry, “Vinča” Institute of Nuclear Sciences, National Institute of the Republic of Serbia, University of Belgrade, Belgrade, Serbia

**Keywords:** Development, Gene expression, Gene regulation, Cellular signalling networks, Non-coding RNAs

## Abstract

Rare copy number variants (CNVs) are among the most common genomic disorders underlying CAKUT. miRNAs located in rare CNVs represent well-founded functional variants for human CAKUT research. The study aimed to identify and functionally interpret miRNAs most frequently affected by rare CNVs in CAKUT and to estimate the overall burden of rare CNVs on miRNA genes in CAKUT. The additional aim of this study was to experimentally confirm the effect of a rare CNV in CAKUT on candidate miRNA’s expression and the subsequent change in mRNA levels of selected target genes. A database of CAKUT-associated rare CNV regions, created by literature mining, was used for mapping of the miRNA precursors. miRNAs and miRNA families, most frequently affected by rare CAKUT-associated CNVs, have been subjected to bioinformatic analysis. CNV burden analysis was performed to identify chromosomes with over/underrepresentation of miRNA genes in rare CNVs associated with CAKUT. A functional study was performed on HEK293 MIR484^+/-^ KO and HEK293 WT cell lines, followed by the analysis of relative miRNA and mRNA target gene levels. 80% of CAKUT patients with underlying rare CNV had at least one miRNA gene overlapping the identified CNV. Network analysis of the most frequently affected miRNAs has revealed the dominant regulation of the two miRNAs, hsa-miR-484 and hsa-miR-185-5p. Additionally, miR-548 family members have shown substantial enrichment in rare CNVs in CAKUT. An over/underrepresentation of miRNA genes in rare CNVs associated with CAKUT was observed in multiple chromosomes, such as chr16, chr20, and chr21. A significant 0.37 fold downregulation of hsa-miR-484, followed by a notable upregulation of *MDM2* and *APAF1* and downregulation of *NOTCH3* was detected in HEK293 MIR484^+/-^ KO compared to HEK293 WT cell lines, supporting the study hypothesis. miRNA genes are frequently affected by rare CNVs in CAKUT patients. Understanding the potential of CNV-affected miRNAs to participate in CAKUT as genetic drivers represent a crucial implication for the development of novel therapeutic approaches.

## Introduction

Congenital anomalies of the kidney and urinary tract (CAKUT) are a diverse group of developmental defects, occurring in approximately 1:500 liveborn children^1^. In addition to its high incidence, CAKUT is the most frequent cause of pediatric kidney failure^2^. Rare copy number variants (CNVs) and point mutations are the most common genomic disorders underlying CAKUT and collectively explain 20% to 25% of cases in cohort studies^3,4^. However, it is not uncommon that for certain CNVs identified in CAKUT the precise genetic driver is unknown. Thus, the availability of the recently described comprehensive CNV landscape of CAKUT^4^ allows further investigation of genetic elements, beyond the protein-coding genes, located in CAKUT-associated CNVs.

One of the intriguing mechanisms which have the potential to orchestrate genomic regulatory networks is based on microRNAs (miRNAs). Conditional loss of kidney miRNAs induced by the deletion of Dicer, the enzyme responsible for miRNA processing, leads to the formation of CAKUT in mice^5^. However, the research of miRNA role in CAKUT development is currently at its beginnings^6^. Targeted sequencing of 96 kidney developmental miRNAs has shown that point mutations in these miRNA genes do not play major roles in CAKUT^7^. On the other hand, significantly different miRNA expression levels have been found in tissue from patients with CAKUT compared to healthy control tissue^8^. It was shown previously that changes in miRNA gene dosage can dramatically alter their cellular levels and subsequently affect the target gene’s expression in the corresponding individuals^9^. Thus, miRNAs located in rare CNVs represent well-founded functional variants that have to be considered in human CAKUT research. To date, there are no studies comprehensively investigating the potential effects of disrupted miRNA genes by rare CNVs on CAKUT pathogenesis.

The study aimed to perform a miRNA-centric analysis to identify and functionally interpret miRNAs most frequently affected by rare CNVs in CAKUT complemented by a CNV-centric analysis to estimate the overall burden of rare CNVs on miRNA genes in CAKUT. Based on the functional interpretation, the additional aim of this study was to experimentally confirm the effect of a rare CNV in CAKUT on candidate miRNA’s expression and the subsequent change in mRNA levels of selected target genes.

## Materials and methods

### Collection of data about rare CNVs in CAKUT and miRNA mapping

A database of rare, pathogenic and likely pathogenic CNV regions identified in aggregate of CAKUT phenotypes has been populated through literature mining^4,10–14^ (Online resource, Supplementary Table 1). Genomic coordinates have been defined in the hg19 assembly version. Synchronization of CNV regions specified in different hg assemblies with the hg19 assembly was performed using the Liftover function of the UCSC genome browser^15^. Mapping of the miRNA precursors onto collected rare CNV regions was performed using the Table browser tool^16^ of the UCSC genome browser^15^ with the following parameters: clade: mammal; genome: human; assembly: hg19; group: genes and gene predictions; track: sno/miRNA; table: wgRna. The final iteration of miRNA mapping was performed on April 2021, (UCSC Table browser data last updated 2021-02-18).

### Identification of miRNAs most frequently affected by CNVs associated with CAKUT

**Figure 1:**
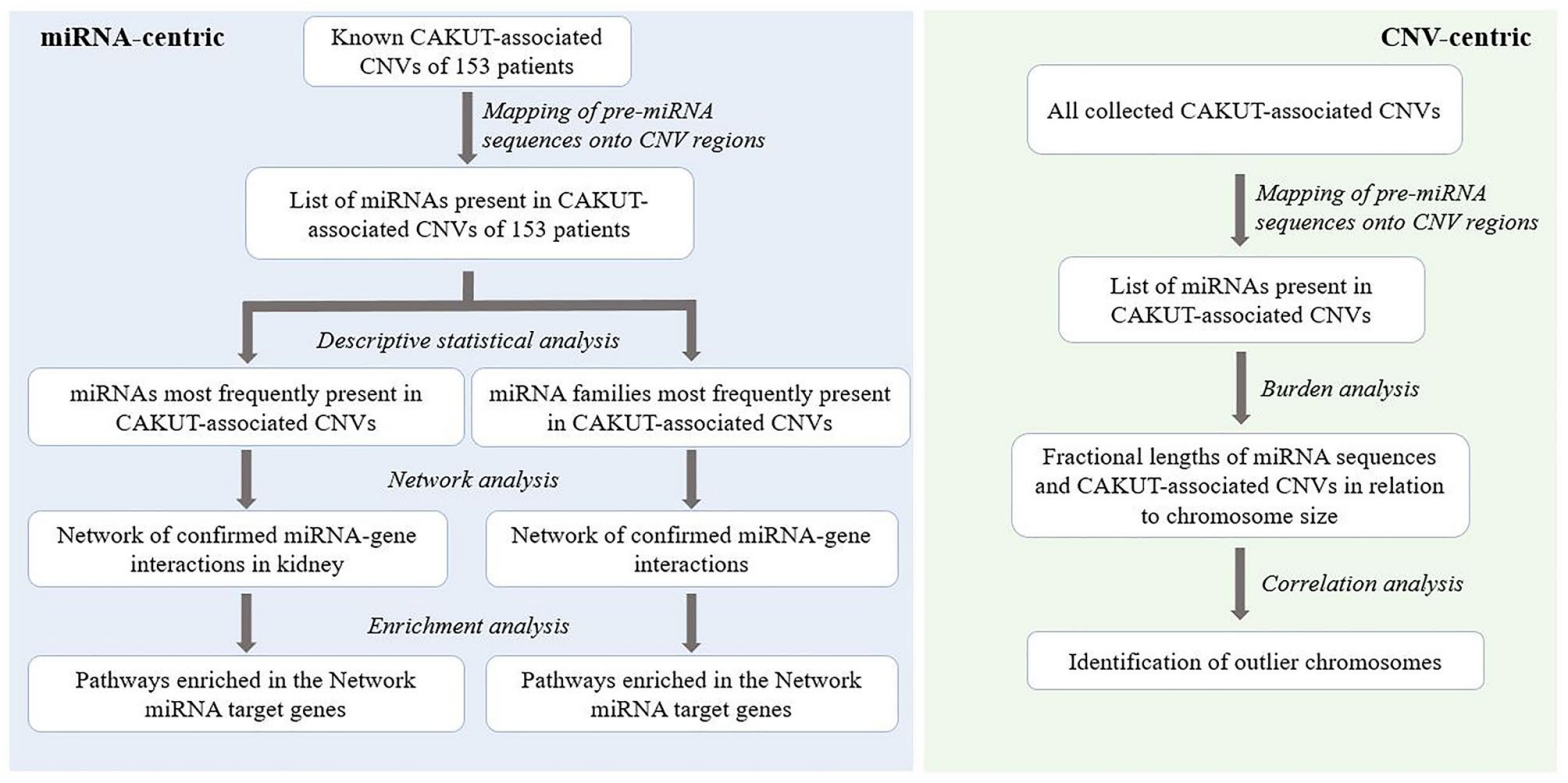
Study design. The miRNA-centric approach was based on data from 153 patients with identified CAKUT-associated CNVs^4^ to functionally interpret the most frequently affected miRNAs. The CNV-centric approach aimed to estimate the overall burden of CAKUT-associated CNVs on miRNA genes, by including all major studies investigating CNVs in CAKUT^4,10–14^.

For the identification of miRNAs most frequently affected by rare CNVs in CAKUT (miRNA-centric analysis), the most comprehensive collection of CAKUT-associated CNVs^4^ was used (Fig. 1). After miRNA mapping, descriptive statistical analysis of the generated database of mapped miRNAs was performed in RStudio 1.3.1093 version with installed R 4.0.3 version, using *sqldf* package to perform SQL selects on R data frames. Knowing that genes of the same miRNA, occupying different genomic locations, and miRNAs from the same family both have annotation suffixes^17^, *stringr* package was employed to subtract miRNA annotation suffixes for an additional miRNA family analysis. This allows the identification of frequently affected miRNA families where different family members are affected in a small number of patients. The results of all performed selects have been exported into an Excel file for further data summary.

### Bioinformatic analysis of the most frequently affected miRNAs by rare CNVs in CAKUT

Top-rated miRNAs and miRNA families, most frequently affected by rare CNVs in CAKUT patients, have been subjected to bioinformatic analysis. The arbitrary cut-off has been placed for the top ten miRNAs most commonly affected by rare CNVs. This cut-off also allows for a more meaningful bioinformatic interpretation of the top-rated miRNAs. For the top-rated miRNAs, a network of miRNA-gene interactions has been created using the miRNet v2.0 tool^18^. The network has been woven for the miRNAs known to be active in kidney tissue, using miRTarBase v8.0 as a resource of validated miRNA-gene interactions. To interpret the network interactions in addition to their visualization, enrichment analysis was performed on target genes, using the Kyoto Encyclopedia of Genes and Genomes (KEGG) database and hypergeometric test as enrichment algorithm^18^. Molecular pathways were considered significantly enriched when adj. P < 0.05. Centrality analysis for the identification of network hubs has been performed using centrality measures expressed in degree and betweenness parameters^18^. For network analysis of miRNA families affected by rare CNVs, network creation has been performed on the families with the greatest shift toward the top CNV-affected miRNAs after annotation suffix removal. Subsequent analysis has been performed as previously described except not specifying the tissue of interest. That way, most of the miRNA family members were retained in the network which gave a more comprehensive insight into miRNA family pathway regulation.

### Overall burden of rare CNVs on miRNA genes in CAKUT

This CNV-centric analysis aimed to investigate the overall burden of rare CNVs on miRNA genes in CAKUT (Fig. 1). For this purpose, all of the CAKUT-associated CNVs from major studies that have been investigating rare CNVs in CAKUT were included^4,10–14^. The analysis has been performed separately for the two types of CNVs, deletions and duplications. Data pre-processing has included the calculation of the following parameters as described by Marrale et al.^19^:

- length of all pre-miRNA in each chromosome (Lmir) and cumulative length of all rare CNVs in CAKUT per chromosome (Lcnv)
- ratio calculation of Rmir (Rmir = Lmir/chromosome length) and Rcnv (Rcnv = Lcnv/chromosome length)
- counts of unique miRNA loci included in distinct and overlapping CNVs in CAKUT

To calculate the cumulative length of all rare CNVs in CAKUT (Lcnv) per chromosome, three possible scenarios were handled. In the first scenario, CNV does not overlap with any other CNV and thus was considered as a whole. In the second scenario, there is an overlap of multiple CNVs. The region of interest is considered between the minimum and maximum of the overlapping regions. In the third scenario, one CNV is completely overlapped with another CNV and thus the coordinates of the larger CNV were included in the equation while the overlapped CNV is ignored. The total cumulative length of CNVs per chromosome (Lcnv) was the sum of corresponding DNA regions defined in the previously described scenarios. Mapping of miRNAs onto these regions gave counts of unique miRNA loci depicting the overall burden of CNVs on miRNA genes per each chromosome in CAKUT (Online resource, Supplementary Table 2).

The correlation analysis between the number of unique miRNA genes overlapping rare CNVs and the fractional length of CNVs in relation to the size of the chromosome (Rcnv ratios) was performed to investigate if the number of unique miRNA genes included in CNVs follows a linear trend as a function of the cumulative length of CNVs. For each chromosome the number of mapped miRNA genes was plotted against the Rcnv ratios, to find the linear best-fit functions and calculate correlation coefficients. The analyses were performed separately for deletions and duplications using the nonparametric Spearman correlation test. Statistical analysis and graph generation were done using Prism v8 software (GraphPad Software, Inc.).

### Cell culture

**Figure 2:**
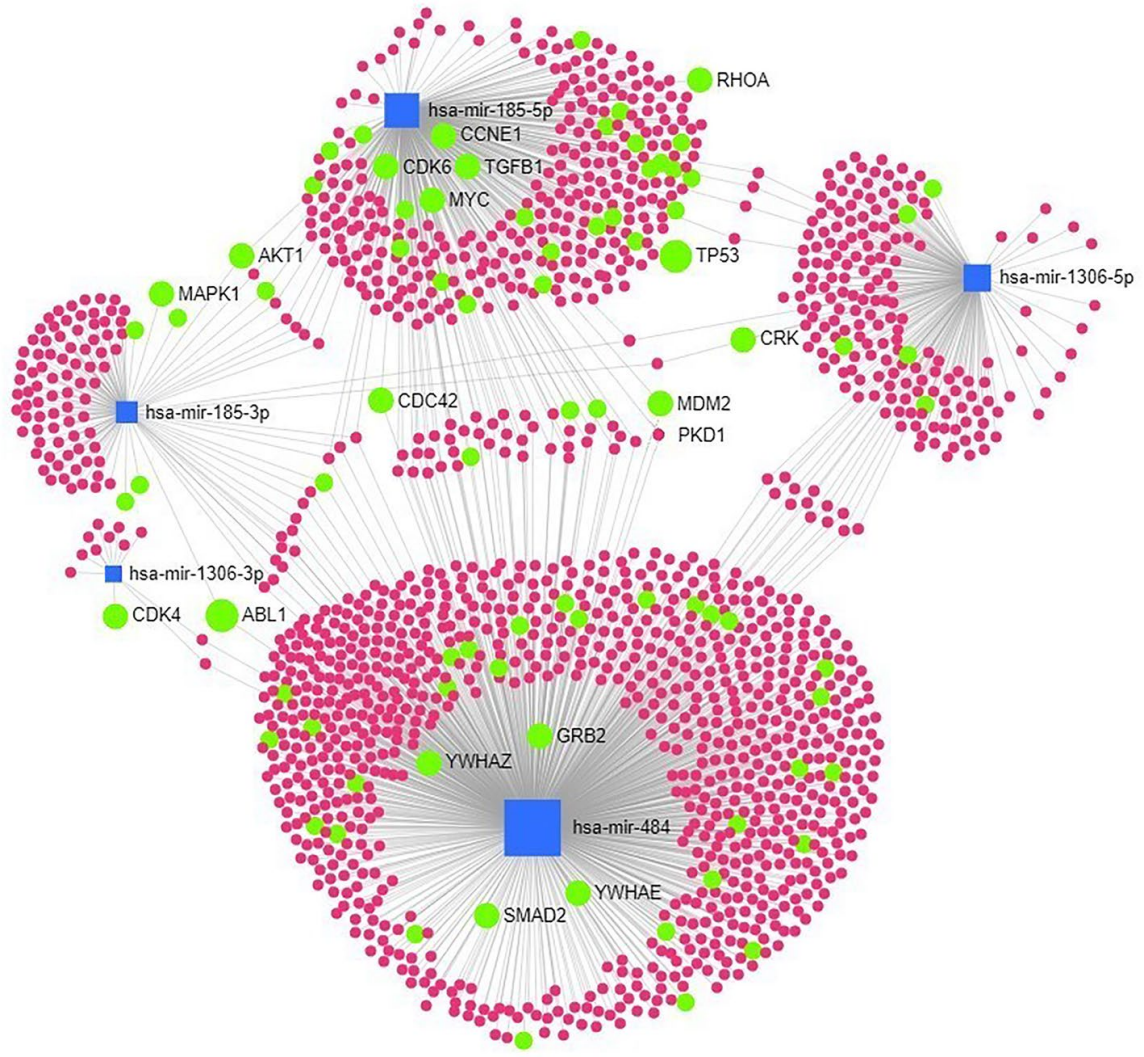
miRNA-gene interaction network of miRNAs most frequently affected by rare CNVs in CAKUT. Only interactions occurring in the kidney tissue have been included in the algorithm. Square shapes represent miRNAs while circles represent genes regulated by specific miRNAs. The size of the squares depicts the number of miRNA-gene interactions based on the miRTarBase v8.0 data. Green circles represent genes included in the significantly enriched pathways (Table 3) whereby the size of the circle is proportional to the number of significantly enriched pathways in which the specific gene co-localises.

In order to achieve the in vitro model which depicts the genomic alteration in CAKUT patients, the heterozygous knockout (KO) of the *MIR484* gene was designed. The hsa-miR-484 was one of the top-rated miRNAs which has generated the largest module in the network analysis (Fig. 2). Additionally, certain target genes of this module have been enriched in significant KEGG pathways (Table 3). The human embryonic kidney cell line (HEK293 WT) and heterozygous MIR484 (GeneID: 619553) knockout HEK293 cell line (MIR484^+/-^ KO) were obtained from Creative Biogene (Shirley, NY, USA). In brief, sgRNA was designed according to the hsa-miR-484 sequence, after which Cas9 and sgRNA vectors were co-transfected into HEK293 cells. Three days after transfection, pool cells were collected to extract genomic DNA to confirm a successful knockdown of hsa-miR-484. The cell pool went through monoclonal growth after which PCR and Sanger sequencing were done for further knockdown validation. Positive MIR484^+/−^ KO clones, with one wild type allele and the second 157 bp deletion allele, were used for further analysis (Creative Biogene, Shirley, NY, USA).

Cell lines were cultured in Dulbecco's modified Eagle's medium (DMEM) with 10% fetal bovine serum (FBS) and 1% penicillin/streptomycin at 37 °C, 95% humidity and 5% CO_2_.

### Extraction of the total RNA from cultured cells

For total RNA extraction, the cell lines were cultured in 25cm^2^ non-filter cap flasks. After the medium was removed and the cells were washed with cold PBS and harvested, total RNA was extracted from three replicates of both cell lines with TRI Reagent^®^ solution (Ambion) according to the manufacturer’s protocol. Extracted total RNA samples were dissolved in the nuclease free water (Ambion) and stored at −80 °C. Total RNA concentration and purity were determined with a NanoDrop ND-1000 spectrophotometer (Thermo Scientific).

### Mature microRNAs expression validation by quantitative real‑time PCR

Generation of cDNA from the total RNA was performed in reverse transcription reaction using TaqMan^®^ microRNA Reverse Transcription kit according to manufacturer’s protocol (Thermo Fisher Scientific Inc., Waltham, USA). The primer pool for reverse transcription consisted of TaqMan^®^ MicroRNA Assay reverse transcription primers for hsa-miR-484 (ID 001821) and *RNU44* (ID 001094) at a final dilution of 0.05×. Synthesized cDNA was stored at −20 °C. The expression levels of hsa-miR-484 were determined by quantitative Real-time PCR on Applied Biosystems Real-Time 7500 system (Applied Biosystems, Inc., Foster City, CA) using the TaqMan microRNA assay: ID 001821 (for hsa-miR-484). Stably expressed reference RNU44 was used as endogenous control to normalize the hsa-miR-484 levels. All reactions were performed in duplicates in a 96-optical well plate at 95 °C for 10 min followed by 40 cycles of 95 °C for 15 s followed by 60 °C for 1 min.

### Relative expression of the hsa-miR-484 target genes by quantitative real‑time PCR

One μg of total RNA was treated with DNAseI (Fermentas, Thermo Fisher Scientific) and the reverse transcription was done using RevertAid First strand cDNA synthesis kit with oligo-dT18 according to manufacturer’s protocol (Thermo Fisher Scientific Inc., Waltham, USA). Expression levels of the selected hsa-miR-484 target genes were measured by quantitative Real-time PCR on Applied Biosystems Real-Time 7500 system (Applied Biosystems, Inc., Foster City, CA) using TaqMan^®^ gene expression assay: Hs00947377_m1 (for *PKD1* (polycystin-1)), Hs00540450_s1 (for *MDM2* (Mouse double minute 2 homolog)), Hs00211420_m1 (for *FIS1* (Mitochondrial Fission 1 Protein)), Hs01128537_m1 (for *NOTCH3* (Neurogenic locus notch homolog)), Hs00559441_m1 (for *APAF1* (Apoptosis protease-activating factor-1)), Hs02786624_g1 (for *GAPDH* (Glyceraldehyde 3-phosphate dehydrogenase)). Relative mRNA levels were normalized using a stably expressed reference gene, *GAPDH*. All reactions were performed in duplicates in a 96-optical well plate at 50 °C for 2 min followed by 95 °C for 10 min followed by 40 cycles of 95 °C for 15 s followed by 60 °C for 1 min.

### Analysis of relative miRNA and mRNA levels

The relative levels of target genes were calculated using the comparative Ct method^20^. The analysis of relative levels of the hsa-miR-484 and its target mRNAs in HEK293 WT and MIR484^+/−^ KO cell lines was performed on 2^−dCt^ values using the Student's t-test. Values of P < 0.05 were considered statistically significant. All analyses and graphical presentation of results were performed using Prism v8 software (GraphPad Software, Inc.).

## Results

### miRNA genes are frequently affected by CNVs in CAKUT

miRNA mapping was performed on a database of pathogenic and likely pathogenic CNVs in CAKUT created by the inclusion of 153 CAKUT patients^4^. CAKUT phenotypes included in the analysis are presented in Table 1. The CNV size ranged from 0.01 to 34.11 Mb. Aneuploidies, although reported for the X chromosome in CAKUT, have been excluded as they were considered as non-informative events regarding functional interpretation. CNVs not encompassing the entire X chromosome have been retained (Online resource, Supplementary table 2, “chr X” sheet). After miRNA mapping, 316 miRNA genes have been identified to be affected by CNVs in CAKUT. miRNA annotation suffix removal has pointed out that these miRNA genes belong to 276 unique miRNAs and miRNA families. CNVs identified in 31 CAKUT patients did not hit any of the miRNA genes.

**Table 1:** Phenotypes of patients, in which rare pathogenic or likely pathogenic CNVs have been identified, included in the estimation of most frequently affected miRNAs.

CAKUT subcategories	Phenotypes	Number of patients
Kidney anomalies (KA)	Renal agenesis, hypoplasia, dysplasia and multicystic dysplasia	99
Obstructive uropathy (OU)	Congenital hydronephrosis, ureteropelvic junction obstruction, ureterovesical junction obstruction and congenital megaureter	17
Posterior urethral valves (PUV)	Posterior urethral valves	9
Vesicoureteral reflux (VUR)	Vesicoureteral reflux	19
Duplicated collecting system (DCS)	Duplications of the ureter or kidney, partial and complete	9

Data extracted from Verbitsky et al., 2019 study^4^.

The top ten miRNAs most frequently affected by CNVs in CAKUT are presented in Table 2. Analysis of miRNA families most commonly affected in patients with underlying rare CNV revealed the enrichment of the miR-548 family (Table 2). As none of the miR-548 family members was among the top ten most frequently affected miRNAs (Table 2), we have consulted the gnomAD SV controls database to compare our results with the impact of polymorphic CNVs on miR-548 family members in controls. Out of the 73 miR-548 family precursors, located across almost all chromosomes, only one precursor, hsa-mir-548i-3 mapped polymorphic CNV in controls. This supports our findings that miR-548 family members show substantial enrichment in rare CNVs in CAKUT. Another enriched miRNA family was miR-378, found in the CNVs of 30 patients (Table 2). hsa-mir-378j was the dominant precursor of this family regarding rare CNV impact, already found as the most frequently affected miRNA before suffix removal (Table 2). The entire data table of mapped miRNA sequences is deposited in Supplementary material (Online resource, Supplementary Table 1).

**Table 2:** The ten miRNAs/miRNA families most frequently affected by rare CNVs in CAKUT patients.

miRNAs ID	N	CAKUT subcategories				
		KA	OU	PUV	VUR	DCS
hsa-mir-378j	24	20	2	1	0	1
hsa-mir-2909	23	19	2	1	0	1
hsa-mir-649	17	13	1	1	1	1
hsa-mir-484	9	6	2	0	0	1
hsa-mir-6506	9	6	2	0	0	1
hsa-mir-5087	8	5	1	1	1	0
hsa-mir-1306	8	5	0	1	1	1
hsa-mir-3680-2	7	3	1	2	1	0
hsa-mir-185	7	5	0	1	0	1
hsa-mir-3618	7	5	0	1	0	1
**miRNA families ID**						
hsa-mir-378	30	26	2	1	0	1
hsa-mir-2909	23	19	2	1	0	1
hsa-mir-649	17	13	1	1	1	1
hsa-mir-548	13	11	0	0	1	1
hsa-mir-484	9	6	2	0	0	1
hsa-mir-6506	9	6	2	0	0	1
hsa-mir-1306	8	5	0	1	1	1
hsa-mir-5087	8	5	1	1	1	0
hsa-mir-185	7	5	0	1	0	1
hsa-mir-3180	7	4	2	0	0	1

N – number of patients harboring rare CNV which contains specific miRNA/miRNA family. CAKUT subcategories: KA – kidney anomalies, OU – obstructive uropathy, PUV – posterior urethral valves, VUR – vesicoureteral reflux, DCS – duplicated collecting system.

### Bioinformatic analysis of miRNAs most frequently affected by rare CNVs in CAKUT patients

For the biological interpretation of miRNAs most frequently affected by rare CNVs in CAKUT patients, a network of miRNA-gene interactions has been created (Fig. 2). Of the ten miRNAs used as an input for network creation (Table 2), only 3 have been retained by the network algorithm allowing only kidney tissue interactions (hsa-mir-484, hsa-mir-185, and hsa-mir-1306). The algorithm has automatically recognized mature forms of miRNAs and their specific interactions. Two major modules characterize the generated network, clustering around hsa-miR-484 and hsa-miR-185-5p. These two miRNAs also have the highest number of intersecting target genes.

Functional annotation of the network genes using hypergeometric test (Fig. 2) has revealed the significantly enriched KEGG pathways (Table 3). It was observed that most of the target genes related to significantly enriched pathways are being regulated by hsa-miR-484 and hsa-miR-185-5p.

**Table 3:** KEGG pathways enriched in target genes of miRNAs and miR-548 family members most frequently affected by rare CNVs in CAKUT.

	Total	Hits	Pval	adj.Pval
**Pathways enriched in target genes of most frequently affected miRNAs**				
Cell cycle	124	23	0.000227	0.0227
mRNA surveillance pathway	82	16	0.00116	0.03275
Pathways in cancer	310	42	0.0012	0.03275
Neurotrophin signalling pathway	123	21	0.00131	0.03275
**Pathways enriched in target genes of miR-548 family members**				
ErbB signalling pathway	87	14	0.000107	0.0098
Dorso-ventral axis formation	12	5	0.000196	0.0098
Adherens junction	70	11	0.000727	0.02135
Regulation of actin cytoskeleton	182	20	0.000854	0.02135

**Figure 3:**
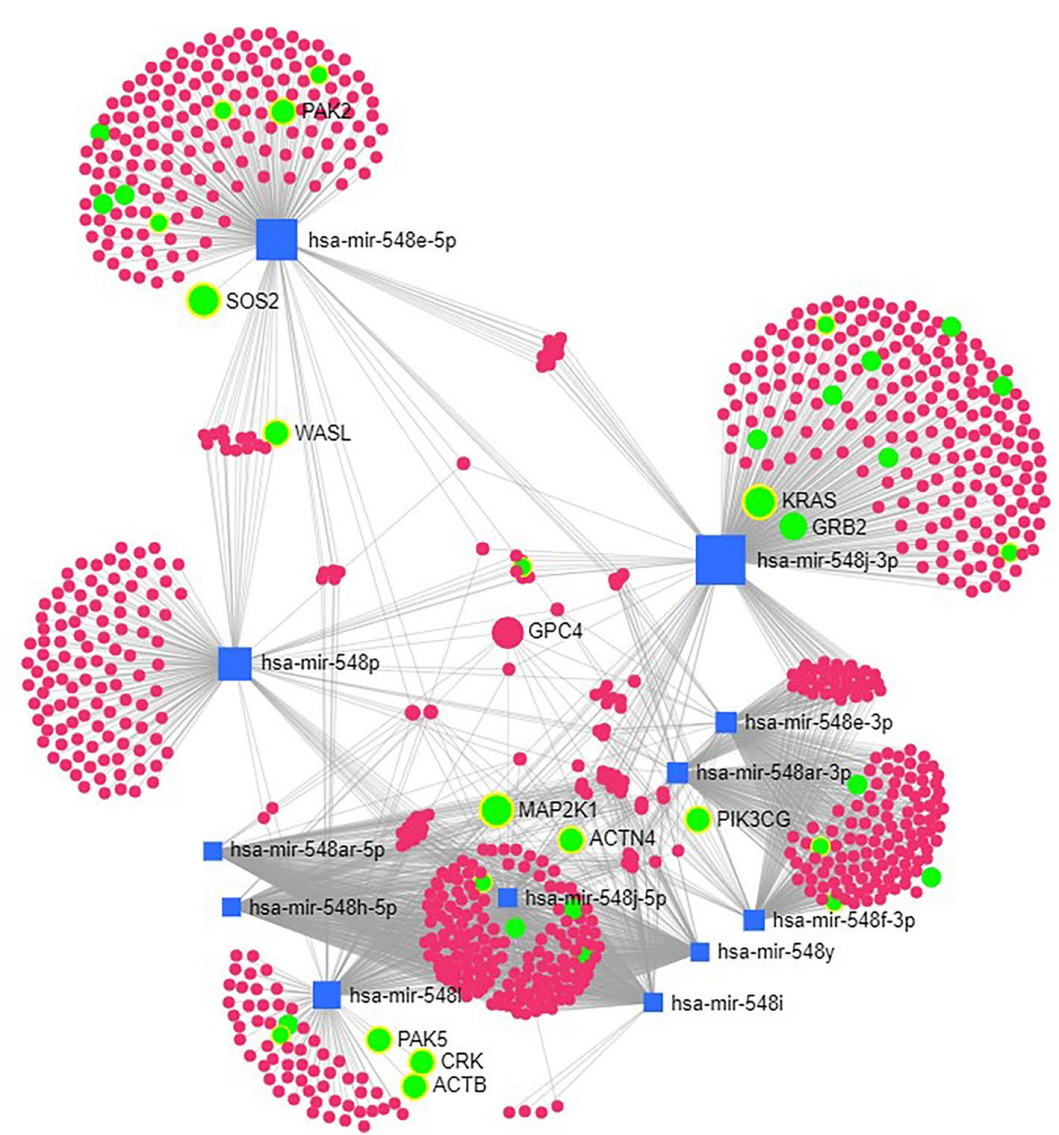
miRNA-gene interaction network of miR-548 family members affected by rare CNVs in CAKUT. Square shapes represent miRNAs while circles represent genes regulated by specific miRNAs. The size of the squares depicts the number of miRNA-gene interactions based on the miRTarBase v8.0 data. Green circles represent genes included in the significantly enriched pathways (Table 3) whereby the size of the circle is proportional to the number of significantly enriched pathways in which the specific gene co-localises. The GPC4 gene, although not involved in significantly enriched pathways, has been emphasized as a gene with the highest centrality, measured in degree and betweenness parameters.

Enrichment analysis was performed on target genes from the miRNA-gene interaction network (Figs. 2 and 3).

Total – number of genes in the pathway; Hits – number of genes in miRNA–gene interaction network involved in the pathway; Pval – hypergeometric test; adj.Pval – adjustment for false discovery rate (FDR).

Due to the observed shift of the miR-548 family toward the top affected miRNA families by rare CNVs in CAKUT (Table 2), we have additionally generated a network of miRNA-gene interactions using miR-548 pre-miRNAs which we have identified to be affected by rare CNVs (hsa-mir-548ar, hsa-mir-548e, hsa-mir-548f-2, hsa-mir-548f-3, hsa-mir-548f-4, hsa-mir-548h-2, hsa-mir-548i-1, hsa-mir-548i-2, hsa-mir-548j, hsa-mir-548l, hsa-mir-548p, hsa-mir-548y). As we aimed to functionally characterize the affected miRNAs from a single family, we have not used filtering for kidney tissue interactions. The algorithm has automatically recognized mature forms of input miRNAs thus including 12 miRNA nodes in the network (Fig. 3). Functional annotation of the network genes using hypergeometric test (Fig. 3) has revealed the significantly enriched KEGG pathways (Table 3). Centrality analysis has pointed to GPC4 as the gene with the highest degree and betweenness parameters, meaning this gene is regulated by the highest number of the network miRNAs from the miR-548 family.

### Relative hsa-miR-484 and target gene mRNA levels

**Figure 4:**
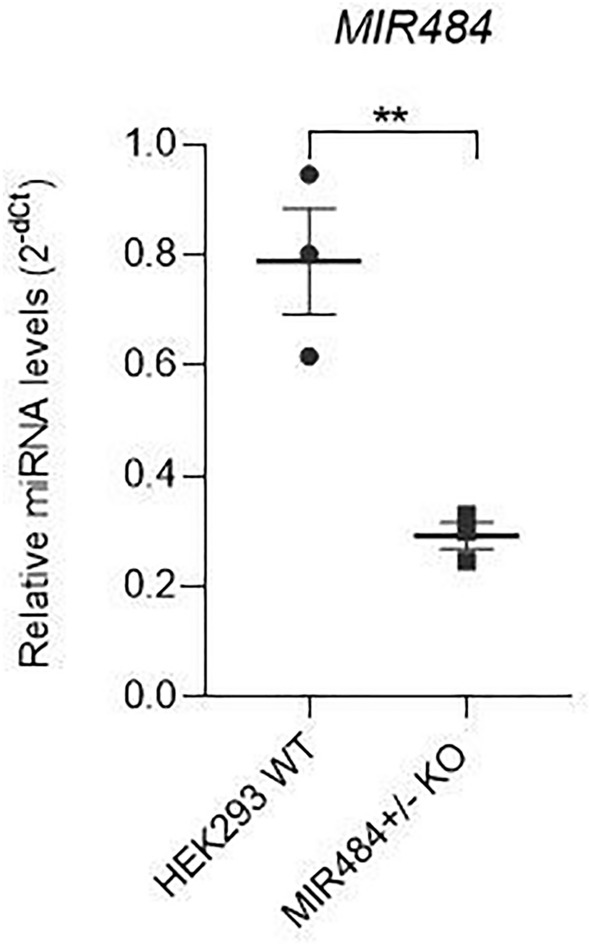
The difference in the relative expression of *MIR484* between MIR484^+/−^ KO and HEK293 WT. Relative miRNA levels were standardized against *RNU44* endogenous control and presented as a scatter plot of 2^−dCt^ values with standard errors of the mean from three independent replicates. The P value was determined using Student's t-test, P = 0.007. ** denotes a significant difference at P < 0.01.

**Figure 5:**
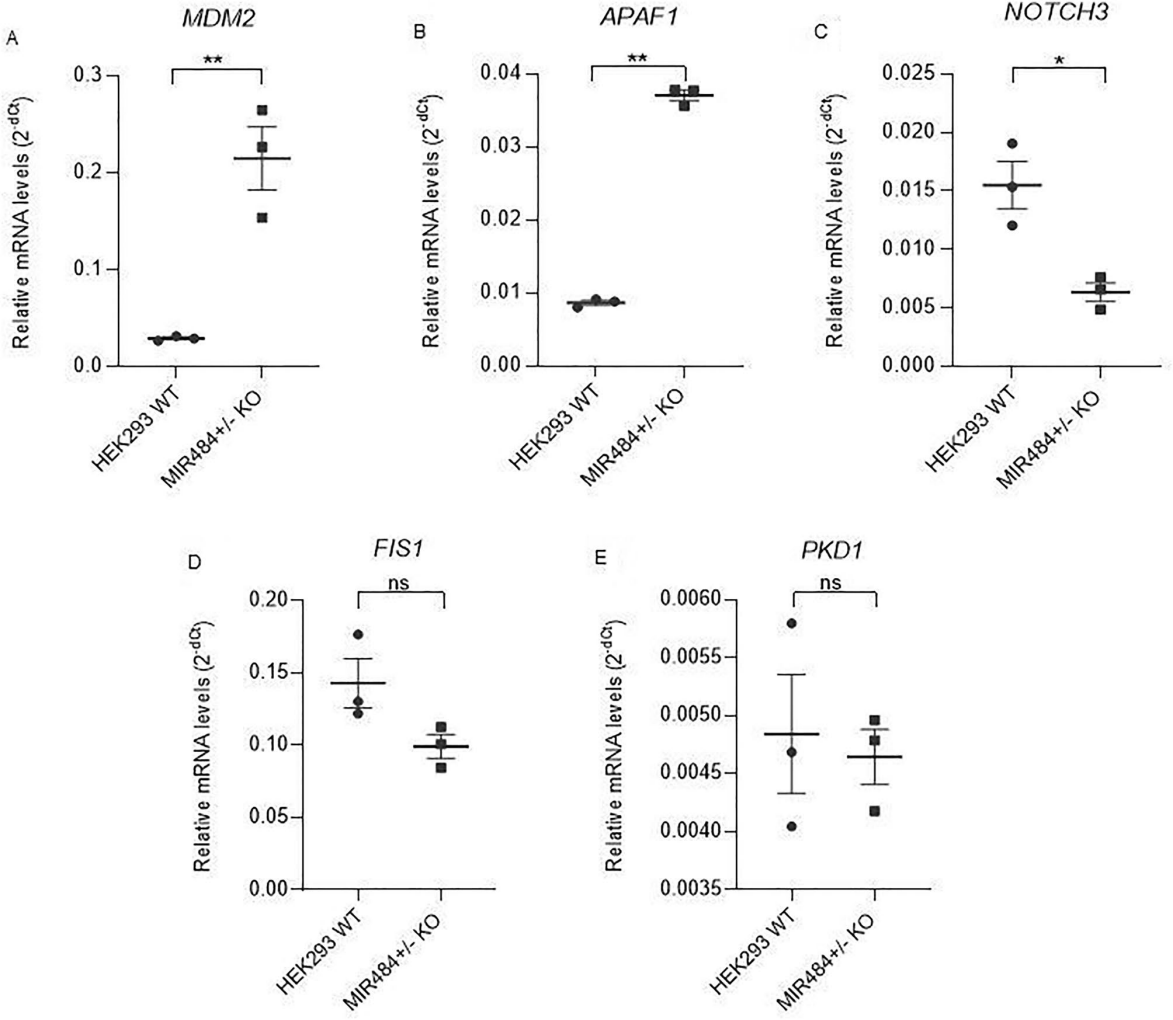
The difference in the relative expression of target genes mRNA between MIR484^+/−^ KO and HEK293 WT. Relative mRNA levels were standardized against *GAPDH* endogenous control and presented as a scatter plot of 2^−dCt^ values with standard errors of the mean from three independent replicates. (**A**) Relative levels of *MDM2* mRNA*,* Student's t-test, P = 0.005. (**B**) Relative levels of *APAF1* mRNA, Student's t-test, P < 0.0001 (**C**) Relative levels of *NOTCH3* mRNA, Student's t-test, P = 0.014. (**D**) Relative levels of *FIS1* mRNA, Student's t-test, P = 0.081. (**E**) Relative levels of *PKD1* mRNA, Student's t-test, P = 0.742. * denotes a significant difference at P < 0.05; ** denotes a significant difference at P < 0.01; ns denotes a nonsignificant difference in relative target gene mRNA levels.

The functional study was performed to experimentally validate the effect of rare CNV on miRNA expression and subsequently the levels of their target genes. We have observed a significant 0.37 fold downregulation of hsa-miR-484 level in MIR484^+/−^ KO compared to HEK293 WT (Student’s t-test, P = 0.007) (Fig. 4). Based on the network analysis and literature research, five candidate target genes with a potential role in CAKUT have been selected for validation of the subsequent effect of CNV-induced hsa-miR-484 downregulation (*MDM2, APAF1, NOTCH3, FIS1* and *PKD1*). We have observed a 7.36 fold upregulation of *MDM2*, 4.28 fold upregulation of *APAF1* and 0.41 fold downregulation of *NOTCH3* in MIR484^+/-^ KO compared to HEK293 WT (Student`s t-test, P_*MDM2*_ = 0.005, P_*APAF1*_ < 0,0001 and P_*NOTCH3*_ = 0.014) (Fig. 5). *FIS1* and *PKD1* did not show a significant difference in mRNA expression levels in MIR484^+/-^ KO compared to HEK293 WT (Student`s t-test, P_*FIS1*_ = 0.081 and P_*PKD1*_ = 0.742 respectively) (Fig. 5).

### Assessment of rare CNV burden on miRNA genes in CAKUT

For assessment of rare CNV burden on miRNA genes in CAKUT, all major studies that have been investigating rare CNVs in CAKUT were included^4,10–14^ (Online resource, Supplementary Tables 1 and 2). Using this approach, an increase in the number of affected miRNAs by CNVs has been observed for certain chromosomes compared to miRNA-centric analysis because of the introduction of the additional CNVs identified in other included studies. We have initially compared the number and length of all known precursor miRNA sequences with the length of the corresponding chromosomes. The fractional length of miRNA genes over the size of the chromosome (Rmir ratio) was of the order of 10^–5^ for all chromosomes except for chromosomes 17 and 19, where the ratio was of the order of 10^–4^ (Table 4). Although the number of identified miRNAs has increased by the time the previous study assessed this parameter^19^ the fraction of the chromosome length covered by microRNA genes still remains very similar for the majority of the chromosomes. On the other hand, the fractional length of CNVs over the size of the chromosome (Rcnv ratios), calculated independently for deletions and duplications (Table 4), substantially differ among the various chromosomes in CAKUT, from a value of 0.0012 up to a value of 0.6691. Additionally, the number of unique miRNA precursors affected by CNVs in CAKUT was also variable across chromosomes. Therefore, a correlation analysis was performed between the number of unique miRNA genes overlapping rare CNVs and the Rcnv ratios to investigate if the number of miRNA genes follows a linear trend as a function of the cumulative length of CNVs.

**Table 4:** Fractional lengths of precursor miRNA sequences and rare CNVs in CAKUT in relation to chromosome size.

Chromosome					Duplication			Deletion	
#	Length (bp)	# of miRNA	Rmir ratio		Rcnv ratio	# miRNA		Rcnv ratio	# miRNA
1	249,250,621	161	5.22E−05		0.0133	4		0.0865	7
2	243,199,373	113	3.73E−05		0.1623	33		0.0341	3
3	198,022,430	93	3.84E−05		0.0113	0		0.1045	13
4	191,154,276	61	2.65E−05		0.0408	6		0.1116	12
5	180,915,260	73	3.38E−05		0.0693	11		0.1395	6
6	171,115,067	67	3.21E−05		0.0086	1		0.0351	1
7	159,138,663	78	4.22E−05		0.0174	3		0.1473	9
8	146,364,022	91	5.01E−05		0.0282	6		0.0471	16
9	141,213,431	84	4.91E−05		0.0240	11		0.0012	0
10	135,534,747	70	4.30E−05		0.0045	0		0.0049	1
11	135,006,516	102	6.02E−05		0.0101	0		0.1934	13
12	133,851,895	76	4.83E−05		0.0589	3		0.0677	1
13	115,169,878	40	2.85E−05		0.0254	1		0.0591	5
14	107,349,540	98	7.43E−05		0.0637	4		N/A	N/A
15	102,531,392	67	5.56E−05		0.0952	10		0.1171	9
16	90,354,753	76	6.71E−05		0.2044	34		0.2170	29
17	81,195,210	108	0.0001077		0.1639	24		0.1415	17
18	78,077,248	35	3.40E−05		0.0193	1		0.0361	0
19	59,128,983	142	0.0001941		0.0037	0		N/A	N/A
20	63,025,520	47	5.95E−05		0.4317	9		N/A	N/A
21	48,129,895	22	3.84E−05		0.0224	3		0.1518	5
22	51,304,566	46	7.15E−05		0.6691	46		0.2016	17
X	155,270,560	118	6.46E−05		0.0101	1		0.0126	2
Y	59,373,566	2	2.34E−06		N/A	N/A		N/A	N/A

miRNA mapping has been performed on all key sources investigating CNV in CAKUT^4,10–14^; Rmir ratio: the ratio between the sum of lengths of all miRNA precursor sequence lengths in a chromosome and the total length of the chromosome; Rcnv ratio: the ratio between the sum of lengths of all rare CNVs in a chromosome and the total length of the chromosome; # miRNA: for each chromosome, the total number of unique miRNA precursor sequences included in all rare CNVs detected in patients.

**Figure 6:**
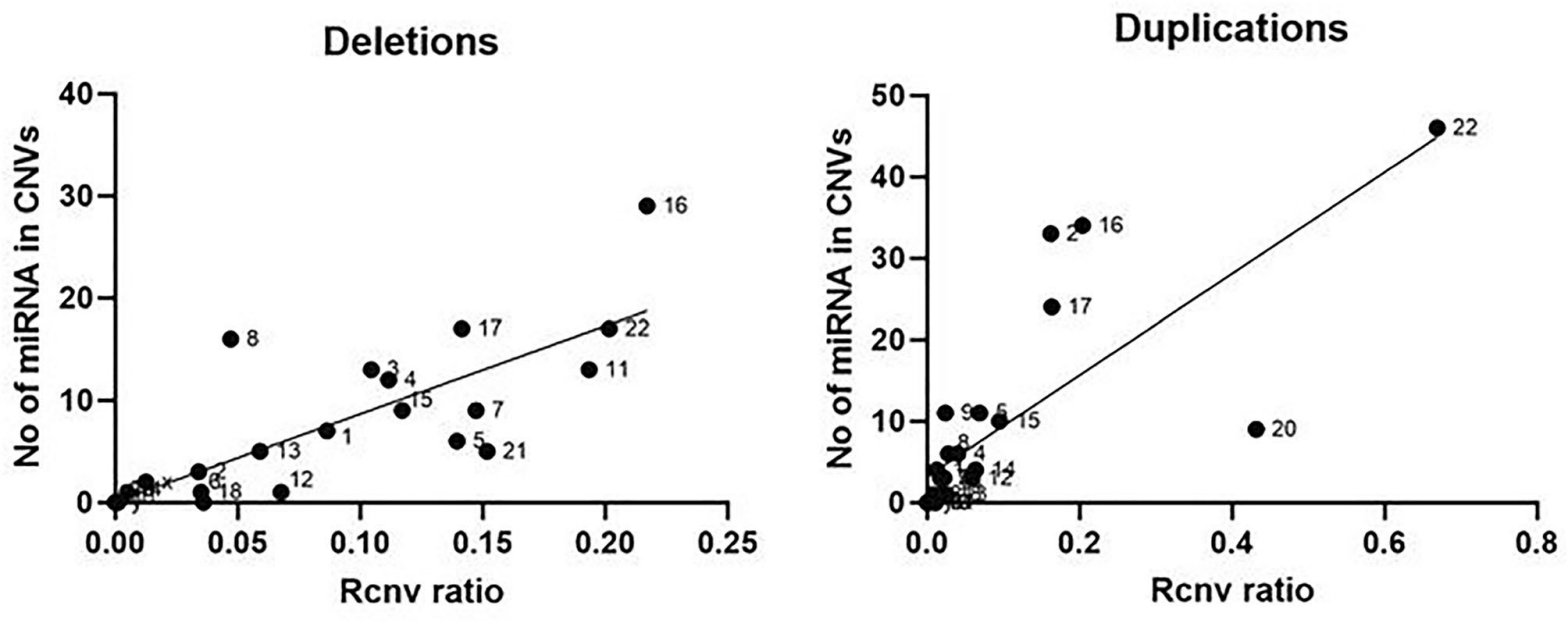
Correlation graph between the number of miRNA genes in rare CNVs and the fractional CNV lengths (Rcnv ratio). For each chromosome, the number of miRNA genes associated with CNVs is plotted as a function of the fractional length of CNV over the chromosome’s size for deletions and duplications, respectively. The graphs depict that most of the data points group close to the best-fit line, indicating a positive correlation between the two variables. However, in certain chromosomes CNVs affect a number of miRNA genes which is higher or lower than expected for the given Rcnv ratio, thus behaving as outliers (correlation analysis was performed on data represented in Table 4).

Calculated values of correlation coefficients between unique miRNA genes overlapping rare CNVs in CAKUT and the Rcnv ratios have shown a statistically significant positive correlation for both deletions and duplications (P < 0.0001, Spearman correlation). It was observed that CNV duplications have slightly higher correlation coefficient (r = 0.8885, 95%CI = 0.7505–0.9522) than deletions (r = 0.8498, 95%CI = 0.6724–0.9349). Rare CNV duplications were found in all chromosomes except Y in CAKUT patients whereas CNV deletions have not been found in chromosomes 14, 19, 20, and Y. Certain chromosomes do not follow the linear trend and are characterized by a relatively large or relatively small number of miRNA genes encompassed with different types of rare CNVs associated with CAKUT (Table 4, Fig. 6). For example, chromosome 16, for both rare CNV deletions and duplications, appears as an “outlier”, which shows a deviation from the best fit line since it includes a much higher number of unique miRNA genes in CNVs. Contrarily, for chromosomes 21 and 20 a deviation from the best fit line could be observed showing a lower number of unique miRNA genes in rare CNV deletions and duplications, respectively.

## Discussion

In this study, we have performed a comprehensive analysis of miRNA genes affected by known, rare CNVs associated with CAKUT. Two complementary approaches have been employed to identify and functionally interpret the most frequently affected miRNAs (miRNA-centric approach) and to estimate the overall burden of rare CNVs on miRNA genes in CAKUT (CNV-centric approach). In addition, by designing in vitro model which depicts the heterozygous deletion of the *MIR484*, we have functionally validated the study concept by confirming the effect of rare CNVs on miRNA expression and subsequently the dysregulation of selected hsa-miR-484 target genes.

The miRNA-centric analysis has pointed out that miRNA genes are frequently affected by rare CNVs in CAKUT. Our study showed that nearly 80% of CAKUT patients had at least one miRNA gene overlapping identified rare CNV. Although the study used for miRNA-centric analysis encompasses ~ 3000 patients^4^, CAKUT phenotypes were not equally represented. This limitation should be addressed in future studies in which an independent replication cohort will be employed. Still, seven out of ten most frequently affected miRNA genes identified in our analysis were previously reported in CAKUT CNVs, on smaller sample size (*MIR484, MIR185*, *MIR6506, MIR2909, MIR378j, MIR3618, and MIR1306*)^13^ supporting the hypothesis of their potential role in CAKUT. The additional complementary CNV-centric analysis has pointed out that in certain chromosomes there was an overrepresentation or underrepresentation of miRNA genes in rare CNVs associated with CAKUT. These CNVs could be of particular interest for future studies investigating how the number of miRNA genes in specific rare CNVs could affect CAKUT phenotype variability. Network analysis of the most frequently affected miRNAs, filtered for kidney tissue interactions, has revealed the dominant regulation of the two miRNAs, hsa-miR-484 and hsa-miR-185-5p, and enrichment of general developmental pathways known to be implicated in CAKUT development. To investigate molecular mechanisms in CAKUT which involve miRNAs in CAKUT-associated CNVs we performed a functional study. We have selected hsa-miR-484 for validation as one of the most frequently affected miRNAs by rare CAKUT-associated CNVs. This miRNA has generated the largest module in the network analysis harboring target genes enriched in significant KEGG pathways (Table 3). It was confirmed through the functional study that rare heterozygous deletions affecting the *MIR484* gene, as identified in CAKUT patients, could substantially downregulate hsa-miR-484 levels in vitro. Additionally, certain target genes of the proposed miRNA showed remarkable dysregulation in cells with *MIR484* heterozygous deletion, which suggests that these events could have a substantial effect on CAKUT penetrability and expressivity and even to be proposed as a potential genetic driver in unresolved rare CNVs in CAKUT.

Five candidate target genes with a potential role in CAKUT have been selected for validation of the subsequent effect of CNV-induced hsa-miR-484 downregulation. We confirmed that reduced expression of hsa-miR-484 is associated with significant upregulation of *MDM2,* which is also suggested by the network analysis. MDM2 is a master negative regulator of tumour suppressor gene P53^21^. p53^−/−^ mouse embryos exhibited a spectrum of congenital abnormalities of the kidney and urinary tract^22^. Additionally, MDM2 overexpression could cause cell death in a p53-independent manner^23^. Besides its role in cell cycle surveillance MDM2 also exhibit pathophysiological functions in inflammation and fibrosis^24,25^, which are major processes in CKD. NF-κB was previously shown to induce MDM2 expression^26^. Upregulation of MDM2 is also observed in the tubulointerstitium of patients with tubulointerstitial fibrosis and unilateral urethral obstruction (UUO) mice^25^. Regulation of MDM2, at the level of transcription, translation, and protein modification, can be very intricate in the context of the therapeutic outcome and is essential to understand p53 dependent and independent activities of MDM2^27^.

The Notch signalling pathway is also involved in kidney development, having a critical role in the early stages of nephrogenesis^28^. Upregulation of *Notch3* in the kidneys of mouse models for PKD has been documented^29^. On the other hand, research on Notch3 knock-out (Notch3^−/−^) mice showed that Notch3 deficiency has an important impact on renal hemodynamics^30^. In our study, the observed downregulation of hsa-miR-484 was associated with the downregulation of *NOTCH3* which should be further considered in the context of CAKUT progression*.* Recently, interaction and regulatory mechanisms between MDM2 and NOTCH1 have been suggested in renal fibrosis^25^. The authors observed attenuation of Notch1 signaling during fibroblast activation mediated by MDM2 and thus suggest that NOTCH1 degradation is one of the downstream mechanisms of MDM2 profibrotic property in the kidney^25^. Thus, future functional studies should further clarify the potential role of hsa-miR-484 in renal fibrosis and mechanisms of interaction and regulation between MDM2 and NOTCH signalling, which may represent potential pharmacological targets for prevention and treatment.

The *APAF1*, which was another gene investigated hereby, has a central role in the apoptosis pathway. APAF1 protein expression is a limiting factor in apoptosome formation and apoptosis signaling^31,32^. Present work unveils that reduction in the expression level of hsa-miR-484 in MIR484^+/-^ KO cells is associated with the upregulation of *APAF1*. Its upregulation could possibly affect apoptosis during kidney development and disturb normal renal development. Recently, it has been demonstrated that apart from its role in apoptosis, APAF1 has a role in centrosome stability^33^. The centrosome is important for cell division, cell migration and regulation of cell cycle events, which were also observed to be enriched in the network genes of frequently affected miRNAs. Increasing centrosome number in vivo perturbed proliferation and differentiation of renal progenitors, resulting in defective branching morphogenesis and renal hypoplasia^34^. Therefore, the upregulation of *APAF1* could have a crucial role in renal development. Recently requirement of centrioles to restrain p53-mediated apoptosis has been documented in lung branching and endoderm development suggesting a link between centrioles and apoptosis^35^.

FIS1 is involved in mitochondrial fission, shaping mitochondrial morphology, and apoptosis^36,37^. Renal inflammation and tissue damage during acute kidney injury and chronic kidney disease have been linked to mitochondrial structural and functional alterations^38,39^. Also, the requisites for intestinal secretory cell differentiation are shown to be a decreased mitochondrial respiration by FOXO/Notch signalling followed by increased mitochondrial fission, triggered by the miR-484-mediated upregulation of FIS1^40^ which highlights the importance of mitochondria in determining stem cell fate. Although there is evidence that hsa-miR-484 targets *Fis1* mRNA^41^ we did not observe the difference in *FIS1* mRNA levels in MIR484^+/−^ KO compared to HEK293 WT. The reason for not detecting the changes on the mRNA level could be due to the specific hsa-miR-484 regulation of FIS1 protein expression by binding to the amino acid coding sequence of Fis1 mRNA^41^ and thus inhibiting its translation and reducing only the protein abundance. Additionally, as FIS1 is required for lowering mitochondrial reactive oxygen species and restoring metabolic homeostasis^42^ it is expected to be regulated by multiple factors.

By observing the network of the most frequently affected miRNAs, it could be deduced that both hsa-miR-484 and hsa-miR-185-5p regulate *PKD1*, a well-known autosomal dominant genetic driver of PKD^43^. Intriguingly, *PKD1* either underexpressed^44,45^ or overexpressed^46,47^ has been shown to cause PKD in animal models. It was thus proposed that a balanced level of functional PKD1 protein is important to maintain tubular architecture^48^. Although *PKD1* could have a possible role in the development of CAKUT^49^, we did not notice the difference in *PKD1* mRNA levels in MIR484^+/−^ KO compared to HEK293 WT. By taking into account the previously described regulation of the FIS1 which is depicted in the inhibition of translation^41^ there is a need for further functional studies to clarify the role of *PKD1* in CAKUT regarding hsa-miR-484 downregulation.

Our findings have demonstrated that the CNV-induced downregulation of the hsa-miR484 is associated with *MDM2*, *APAF1* and *NOTCH3* expression dysregulation, which could potentially influence kidney development*.* This warrants the translational potential of miRNAs, which are observed to be frequently affected by rare CNVs in CAKUT. Further studies are required to adequately determine the mechanisms by which MDM2, APAF1 and NOTCH3 could act in CAKUT, either by leading to urinary system malformation or by activating inflammation and fibrosis-associated processes as the main contributors in kidney function loss.

The analysis of the most frequently affected miRNA families in CAKUT-associated CNVs has pointed toward notable enrichment of the miR-548 family. The hsa-miR-548 family is a large miRNA family specific for primates, and it consists of 73 precursors transcribed from almost every human chromosome (miRBase Release 22.1)^50^. Interestingly, while the members of this miRNA family are relatively highly affected in CAKUT, it is almost completely unaffected by polymorphic CNVs found in controls, regardless of their high number and chromosomal dispersion, which suggest evolutionary pressure on CNVs encompassing hsa-miR-548 family genes. This speculation could also be supported by the functional enrichment analysis of the hsa-miR-548 family target genes, depicting key developmental processes. The major hub of the hsa-miR-548 family interaction network (GPC4) is shown to be an important factor in branching morphogenesis mediated by hepatocyte growth factor HGF^51^. It was shown that GPC4 expression increases during nephrogenesis, which makes it important for the HGF to exert a significant morphogenic effect on the tubule, thus potentially regulating the late stages of tubule formation^52^. The dysregulation of the hsa-miR-548 family members caused by rare CNVs in CAKUT is therefore warranted to be investigated in future studies.

Over the last years, both CNVs and microRNAs come up as potentially important molecular factors involved in CAKUT aetiology. However, in most of the studies investigating the molecular basis of CAKUT development, these two topics have been explored separately. The translational capacity of miRNA to be employed in therapeutic approaches is nowadays increasingly investigated. Numerous preclinical studies utilizing various disease models have tested the use of these new-generation therapeutics, and several miRNA-based therapeutics have advanced into clinical testing^53^. Therefore, the untangling of the mechanisms affected by dysregulated miRNAs could serve for future extension of genetic testing and the development of novel miRNA targeting strategies in CAKUT.

## Supplementary Information

Supplementary Tables.

## Data Availability

All data generated or analysed during this study are included in this published article (and its supplementary information files).
